# Congenital hemophilia A with low activity of factor XII: a case report and literature review

**DOI:** 10.1186/s13052-021-01137-x

**Published:** 2021-10-11

**Authors:** Baoyu Lei, Chuang Liang, Haiyan Feng

**Affiliations:** Department of Neonatology, Maoming People’s Hospital, Weimin Road, Maonan District, Maoming, 525000 Guangdong China

**Keywords:** Congenital hemophilia A, Factor VIII, Factor XII, Inhibitor, Neonate

## Abstract

**Background:**

Congenital hemophilia A is a recessive inherited hemorrhagic disorder. According to the activity of functional coagulation factors, the severity of hemophilia A is divided into three levels: mild, moderate and severe. The first bleeding episode in severe and moderate congenital hemophilia A occurs mostly in early childhood and mainly involves soft tissue and joint bleeds. At present, there are limited reports on severe congenital hemophilia A with low factor XII (FXII) activity during the neonatal period.

**Case presentation:**

A 13-day-old neonate was admitted to the hospital with hematoma near the joints of both upper arms. Coagulation tests showed he had low activity of factor VIII (FVIII) and FXII. He was diagnosed with congenital hemophilia A and treated with human coagulation factor VIII (recombinant FVIII). Although the hematoma became smaller, FVIII activity was only increased to a certain extent and FXII activity decreased gradually. Unfortunately, the child responded poorly to recombinant human coagulation factor VIII and his guardian rejected prophylactic inhibitors and genetic testing and refused further treatment. Three months later, the child developed intracranial hemorrhage (ICH) due to low FVIII activity.

**Conclusions:**

In hemophilia A, the presence of FVIII inhibitors, drug concentration and testing are three important aspects that must be considered when FVIII activity does not reach the desired level. Early positive disease treatment and prophylaxis can decrease the frequency of bleeding and improve quality of life. We recommend that pregnant women with a family history of hemophilia A undergo early prenatal and neonatal genetic testing.

## Background

Congenital hemophilia A is an inherited hemorrhagic disorder caused by the x-linked chromosome factor VIII (FVIII) and it occurs in 0.01% of newborns. Thirty percent of these patients have spontaneous mutations in FVIII and do not have a family history of hemophilia A [1, 2]. In an observational cohort study consisting of 679 patients with severe or moderate hemophilia A, the researchers found that the first bleeding episode in hemophilia A occurred at the median age of 0.82 years in severe disease and 1.47 years in moderate disease [3]. Acute joint hemorrhage is a common symptom. Patients often suffer from repeated bleeding and chronic joint injury [4]. In addition, in 1–4% of patients, intracranial hemorrhage could be the first symptom [5]. When the FVIII activity is < 1%, repeated episodes of spontaneous bleeding occur in approximately 50–60% of patients [6]. Primary prevention in patients with severe hemophilia A can reduce the progression of arthropathy [7].

It is found that the lower the FVIII activity in the body, the more frequent bleeding episodes [5]. The severity of hemophilia is classified according to the activity of functional coagulation factors, with 5–40% being mild, 1–5% being moderate and < 1% being severe [8, 9]. Currently, the main prevention and treatment method is to use plasma-derived or recombinant FVIII products [9]. In addition, bleeding episodes could also be treated with activated prothrombin complex concentrate (aPCC) or recombinant activating factor VII (rFVIIa) [10]. Here, we report the case of a very early onset of severe neonatal congenital hemophilia A and perform a brief literature review on its mechanism.

## Case presentation

**Fig. 1:**
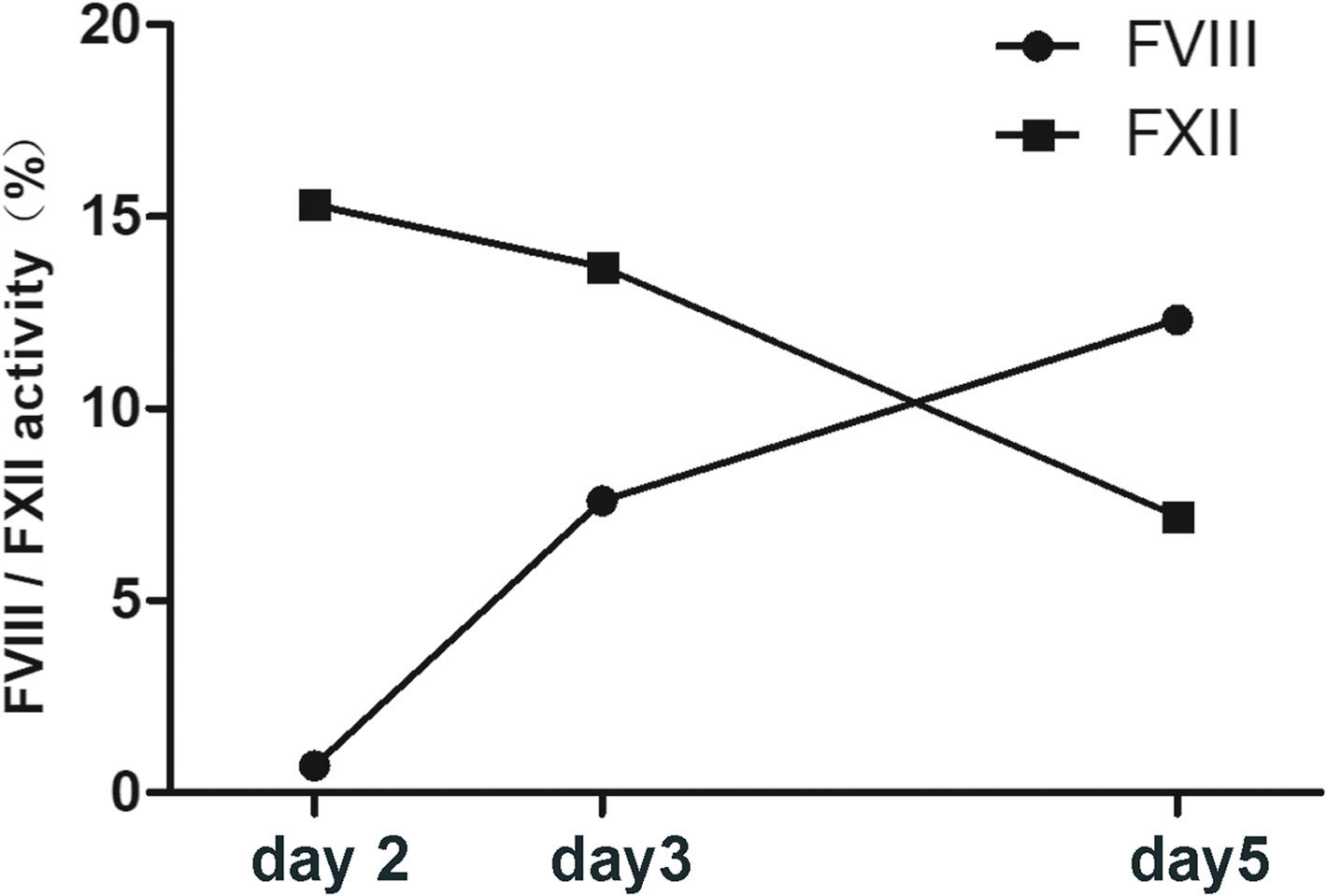
After treatment with human coagulation factor VIII for 5 days, FVIII activity was not significantly increased and FXII gradually decreased

We encountered a 13-day-old neonatal patient with congenital hemophilia A with low FXII activity. At first, he was admitted for hematoma near the joints of both upper arms. Blood test results showed that activated partial thromboplastin time (APTT) was prolonged without extended prothrombin time (PT) and the activity of factors VIII, IX, XI and XII was low, especially the activity of FVIII (0.7%) and factor XII (15.3%). The newborn had a family history of hemophilia A, with an uncle diagnosed with hemophilia A. The neonate was diagnosed with congenital hemophilia A. After admission, he was treated with human coagulation factor VIII with standard dosage according to the guidelines for the management of hemophilia [11, 12]. In general, each unit of FVIII/kg per 8–12 h infused intravenously raises plasma FVIII levels by approximately 2% in the absence of an inhibitor [11, 12]. After 5 days of treatment, the APTT returned to normal and the hematomas became smaller. But the FVIII activity of this patient did not reach the desired level, remaining below 20%, and FXII gradually decreased (Fig. 1). We believed that the plasma factor peak level response was inadequate and that the duration of administration needed to be longer. However, despite the continued risk of bleeding, the family members stopped treatment and refused further prophylactic inhibitors and genetic testing due to financial and other reasons.

**Fig. 2:**
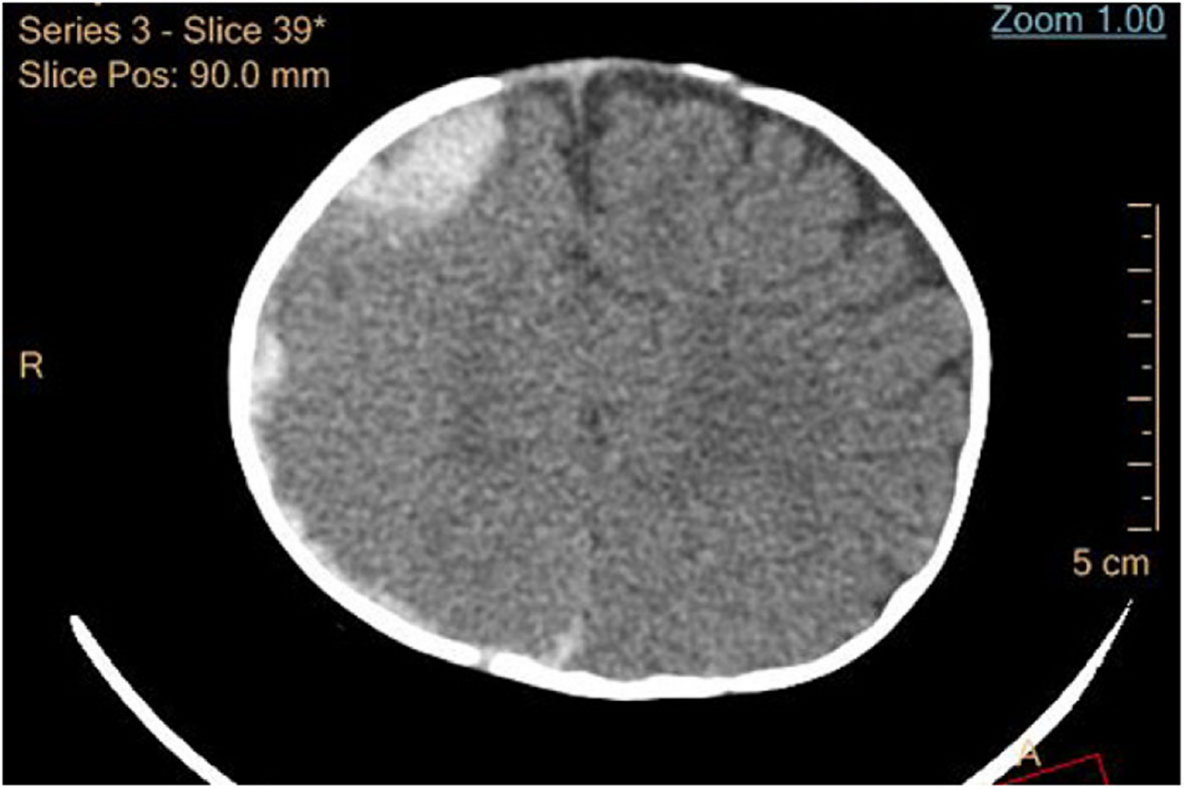
Three months after initial presentation, brain CT scanning showed intracranial hemorrhage

Prophylaxis prevents bleeding and joint destruction and must be initiated 2–3 times per week [11]. Unfortunately, 3 months after all of the treatments were discontinued, he developed convulsions and brain CT scanning revealed intracranial hemorrhage (Fig. 2).

## Discussion and conclusions

Reports of neonatal patients with low FVIII:C and FXII:C are relatively rare. In this case, the neonate’s FVIII activity did not reach the desired level with treatment, and his FXII activity gradually decreased. This case presents some of the challenges of treating patients with hemophilia A. Our patient had severe hemophilia A with FXII deficiency and may have had chromosome and gene mutations. To address these challenges, this paper reviews some new knowledge about congenital hemophilia A and FXII. Currently, three important aspects, namely, the presence of FVIII inhibitors, drug concentration and genetic testing, must be considered when poor treatment response occurs in hemophilia A.

The newborn had low FVIII activity. However, after treatment with human coagulation factor VIII, the activity of FVIII did not increase significantly, as inhibition of FVIII may have occurred. The presence of inhibitors is a common adverse effect of human coagulation factor VIII therapy in patients with hemophilia [13, 14]. Generally, 30 % of patients with severe hemophilia A may develop inhibitors during the first 20 days of exposure to recombinant coagulation factor VIII [15]. The presence of inhibitors may be an immune response to foreign proteins in patients with severe hemophilia A [16]. The inhibitor development depends on the proper activation of antigen-presenting cells (APCs) that encounter FVIII in the periphery, which is a T-cell-dependent process [9]. The total risk of developing inhibitors over a lifetime is 25–40% for severe hemophilia and 5–15% for moderate/mild hemophilia A [16]. However, inhibitors can be produced without previous treatment with human coagulation factor VIII or with only a small amount of blood components [17]. Besides, high doses of recombinant FVIII therapy and surgery may increase the risk of inhibitor development in patients with non-severe hemophilia A [18]. In general, the emergence of inhibitors increases the risk of progressive and disabling joints disease. The sensitive inhibitor screening or the Nijmegen modification method of Bethesda should be used for screening [14, 19, 20].

Despite the potential for the production of inhibitors, the benefits of recombinant coagulation factor VIII still seem to outweigh the risks [21], and better treatment with recombinant FVIII product is possible. Studies have shown that patients treated with factor VIII containing von Willebrand factor (VWF) have a lower incidence of inhibitor production than patients treated with recombinant FVIII product [17]. This may be because the von Willebrand factor obscures the inhibitor epitope in the concentrate, resulting in a longer half-life of the product [14]. Another study suggested that when patients with hemophilia A had inhibitors, clinicians could initiate an immune tolerance induction (ITI) protocol to reduce levels of the inhibitor [14, 22]. A randomized trial showed that ITI eliminated anti-FVIII alloantibodies in about two-thirds of patients [23].

Elimination of inhibitors is important because some asymptomatic patients remain at risk of severe bleeding or life-threatening conditions until the inhibitors are eliminated. Prednisolone has been reported to achieve a complete immunosuppressive response (CR) in some patients [14]. Recombinant FVIII concentrates that produce fewer inhibitors are under study, and a new treatment option for hemophilia patients with inhibitors is the bispecific monoclonal antibody emicizumab [5].

Research findings have shown that plasma-derived or full-length recombinant FVIII have a half-life of between 6 and 25 h [24]. A regimen of lower doses of prophylaxis given more frequently may be an effective option to decrease the frequency of bleeding, joint disease and intracranial hemorrhage. Recombinant FVIII, which effectively prevent spontaneous bleeding, must be injected intravenously three times a week or every other day to maintain FVIII levels ≥1% in patients with severe hemophilia A [25, 26]. Weight-adjusted clearance (CL) of FVIII is related to age and weight. From infancy to adulthood, CL decreases with age and/or weight, and the terminal half-life increases accordingly [27]. Extended plasma half-life FVIII products can produce higher FVIII plasma levels and reduce the number of intravenous injections, which increase the possibility of a more active lifestyle [28]. Currently, half-life extension technology for Fc-fusion proteins or modification with polyethylene glycol (PEG) can prolong the plasma half-life of FVIII, as with efmoroctocog alfa and BAX 855 [26, 29]. Coagulation FVIII produced by Fc fusion technology has few adverse effects because the components of the fusion peptide are plasma proteins, causing fewer allergic reactions [25].

Accurate testing of FVIII is critical for guiding clinical treatment. The test results for coagulation factors are greatly affected by laboratory test methods, and the reason for variation between laboratories is not the bias of instrument calibration, but the differences in reagents, instruments used and test design. The following sampling protocol is recommended for more effective detection of inhibitors: FVIII samples were taken at pre-dose, 15 min, 30 min and at 3, 6, 9, 24, 28 and 32 h post-dose administration to obtain more test information [14]. Results of experiments have shown that test designs for three samples produce more stable results than designs that test only one or two of the diluents. It is recommended that at least three different sample diluents be used in each FVIII:C (FVIII activity) assay with a commutable lyophilised FVIII:C calibrator, which results in a limited reduction of the inter-laboratory variation [30]. However, there are no comparable data to reliably predict an individual patient’s FVIII:C level to guide clinical treatment. Current studies have shown that adults and adolescents need less FVIII/kg than young children to maintain serum drug concentrations. Personalized drug delivery is therefore more suitable in clinical practice [24].

Activated factor XII (Hageman factor, FXII) can trigger the internal coagulation pathway [31], which is measured by APTT [32]. Hageman factor deficiency is usually an autosomal recessive disorder but can be autosomal dominant. Matsushita et al. reported a female patient with hemophilia with an FXII deficiency who had an extremely inactivated normal X chromosome [33]. The exact prevalence of Hageman factor deficiency is not known because patients are normally asymptomatic. Hageman factor deficiency is usually detected by chance in coagulation assay results that isolated prolonged APTT or unexplained coagulation disease [32, 34].

FXII plays an important role in the coagulation system. FXII respectively drives the contact system to initiate coagulation and inflammation through the intrinsic coagulation pathway and the bradykinin-producing kallikrein-kinin system [35]. Humans and animals with low FXII activity have a normal hemostatic ability, but animal models show that FXII is involved in the thrombotic process [36]. FXII was associated with thromboembolic complications, but it was only rarely associated with severe hemorrhagic disease [32].

FXII activity is generally lower in Asians. Deficiency of FXII is an autosomal recessive disorder, but few cases have been reported. Alleles in homozygotes or complex heterozygotes are associated with very low FXII activity (< 1%) compared to unaffected individuals [34]. The autosomal recessive genetic diseases can be prevented by avoiding intermarriage, which requires counselling and education [32]. The average FXII activity depends on the race of the person. One study showed that 95% of healthy Chinese subjects had FXII activity between 47 and 160.25%, and identified some mutations associated with low FXII activity [34].

In addition, results of the coagulation assays are highly age-dependent and must be used to ensure the correct evaluation of coagulation assays in children, especially in the first year of life [37]. For newborns, coagulation factors are already low and gradually increase to adult levels after 6 months [38–40]. On the whole, the low FXII:C in hemophilia A is related to race and age, which is determined by chromosomal and genetic testing.

Unfortunately, the newborn described in this report had a family history of hemophilia A, and the child’s mother did not receive a prenatal genetic diagnosis, nor did she agree to have the child tested for inhibitors and genetic mutations as soon as possible after birth. In one study, Chen et al. developed a noninvasive prenatal diagnosis (NIPD) method for Hemophilia A by sequencing a small target region [41]. A genetic diagnosis can help couples at risk of hemophilia reduce their anxiety about childbirth. The obstetrician must discuss the birth plan with the expectant mother [5]. Hemophilia A is diagnosed by informational gene tracking and/or measurement of fetal FVIII: C level [42]. Determination of a woman’s genetic and phenotypic status before pregnancy is optimal so that she can understand her options and the requirements for a safe delivery [43–45].

In conclusion, this paper presents the case of a newborn with severe neonatal congenital hemophilia A with FXII deficiency. This case highlights the importance of FVIII inhibitors, serum recombinant FVIII concentration and testing in hemophilia A. Prophylaxis treatment is an effective option for decreasing the frequency of bleeding and improving quality of life. We suggest that expectant mothers identified as congenital hemophilia A gene carriers test their fetuses for the FVIII or FXII activity levels and that newborns undergo genetic testing as soon as possible after birth to assess for the risk of the disease.

## Data Availability

All of the data presented in this article can be found in our hospital.

## Abbreviations

FVIII: Factor VIII
ICH: Intracranial hemorrhage
aPCC: Activated Prothrombin complex concentrate
rFVIIa: Recombinant activating factor VII
FVIII:C: FVIII activity
FXII: Factor XII
FXII:C: FXII activity
VWF: Von Willebrand factor
CR: Complete remission
CL: Weight-adjusted clearance
PEG: Polyethylene glycol
APTT: Activated partial thromboplastin time
PT: Prothrombin time
FXIIa: Free factor XIIa
NIPD: Noninvasive prenatal diagnosis
